# Medical physics practice guideline 4.b: Development, implementation, use and maintenance of safety checklists

**DOI:** 10.1002/acm2.13895

**Published:** 2023-02-04

**Authors:** Leigh Conroy, Jacqueline T. Faught, Erika Bowers, Gillian Ecclestone, Luis E. Fong de los Santos, Annie Hsu, Jennifer Lynn Johnson, Grace Gwe‐Ya Kim, Naomi Schechter, Leah K. Schubert, David A. Sterling

**Affiliations:** ^1^ Princess Margaret Cancer Centre Toronto Canada; ^2^ St. Jude Children's Research Hospital Memphis Tennessee USA; ^3^ GenesisCare USA Fort Myers Florida USA; ^4^ Tom Baker Cancer Centre Calgary Canada; ^5^ Mayo Clinic Department of Radiation Oncology Rochester Minnesota USA; ^6^ Odette Cancer Centre Sunnybrook Health Sciences Centre Toronto Canada; ^7^ Potentia Partners Kelburn New Zealand; ^8^ University of California San Diego California USA; ^9^ University of Southern California Los Angeles California USA; ^10^ University of Colorado School of Medicine Aurora Colorado USA; ^11^ University of Minnesota Medical Center Minneapolis Minnesota USA

**Keywords:** MPPG, practice guideline, safety checklist

## Abstract

The American Association of Physicists in Medicine (AAPM) is a nonprofit professional society whose primary purposes are to advance the science, education, and professional practice of medical physics. The AAPM has more than 8000 members and is the principal organization of medical physicists in the US. The AAPM will periodically define new practice guidelines for medical physics practice to help advance the science of medical physics and to improve the quality of service to patients throughout the US. Existing medical physics practice guidelines will be reviewed for the purpose of revision or renewal, as appropriate, on their fifth anniversary or sooner. Each medical physics practice guideline represents a policy statement by the AAPM, has undergone a thorough consensus process in which it has been subjected to extensive review, and requires the approval of the Professional Council. The medical physics practice guidelines recognize that the safe and effective use of diagnostic and therapeutic radiology requires specific training, skills, and techniques, as described in each document. Reproduction or modification of the published practice guidelines and technical standards by those entities not providing these services is not authorized. The following terms are used in the AAPM practice guidelines: Must and must not: Used to indicate that adherence to the recommendation is considered necessary to conform to this practice guideline. While must is the term to be used in the guidelines, if an entity that adopts the guideline has shall as the preferred term, the AAPM considers that must and shall have the same meaning. Should and should not: Used to indicate a prudent practice to which exceptions may occasionally be made in appropriate circumstances.

## 1 INTRODUCTION

### 1.1 Motivation: The value of checklists

The field of medicine is characterized by highly complex and dynamic processes, where a multidisciplinary team works together using sophisticated imaging, planning, and delivery systems to provide efficient, accurate, and safe patient treatment, often under intense time pressure. As a result of such characteristics, the practice of medicine is susceptible to errors in judgment, errors in communication, lack of compliance with standard operating procedures, as well as workflow inefficiencies. Other complex environments outside of medicine, such as aviation^1^, ^2^ and product manufacturing, have successfully used simple tools to aid in reducing human errors. One of these tools is checklists. Checklists have been extensively validated in medical and non‐medical fields for many years and have proven to be an effective tool in error management. They are a key instrument in reducing the risk of costly mistakes and improving overall outcomes.^3^, ^4^, ^5^, ^6^, ^7^, ^8^, ^9^, ^10^, ^11^ As Atule Gawande so eloquently presented in the “Checklist Manifesto”, we must be realistic regarding the complexity and responsibility of our field, and humble enough to accept the benefits in error‐reduction that checklists provide.^12^ Checklists are only effective if they are used, and used as intended. Checklists should be used in conjunction with professional experience, knowledge, and inquiry. Effective implementation of checklists is dependent on the attitudes and motivation of involved staff and leadership.

### 1.2 Goals

The goal of this document is to provide a comprehensive strategy for designing, implementing, using, and maintaining clear and effective safety checklists. It is also intended to provide standard components of checklists that can be used in the development of procedure‐ and clinic‐specific quality management tools. This document does not define the specific elements of a unique checklist for a specific clinical task or process.

Over the past 5 years, since the original MPPG4a was published, interest in and use of safety checklists in medicine^13^, ^14^ and medical physics^15^, ^16^, ^17^, ^18^ has continued to grow. In this updated document we reinforce the strategies presented in the original document, and further address common barriers to checklist implementation and use in the context of change management.

### 1.3 Scope

Given the wide variety of practices and technologies in diagnostic imaging, nuclear medicine, and radiation therapy, it is neither practical nor desirable in this document to provide a rigid set of checklists that must be adhered to. Experience from the aviation industry indicates that effective checklists are “works in progress” that evolve as techniques develop and technology advances. Additionally, effective checklists should fit the needs, workflow, and goals of a specific environment or practice. This document, therefore, focuses on guidelines for development of checklists, rather than rigid recommendations. Future AAPM Task Groups or accreditation organizations (e.g., ACRO, ACR, or ASTRO) should consider using the steps and methods presented in this document when developing standardized safety checklists as part of their documents.

The scope of this MPPG is limited to:

1. Checklist design and implementation recommendations.
2. Providing a few example checklists and checklist components, (not intended to be adopted en bloc). See Appendix C.
3. Identifying strategies for maximizing use of checklists in the clinical environment.
4. Identifying the necessary cultural and organizational features needed to develop, implement, and maintain effective checklists.^10^, ^19^

### 1.4 Intended users

The intended users of this MPPG are individuals involved in quality and safety management in a clinical setting.

## 2 THE ROLE OF CHECKLISTS IN ERROR MANAGEMENT

Most tasks can be classified into two basic categories, depending on the type of behavior needed for completion: tasks requiring schematic behavior, in other words done reflexively or “on autopilot”, and tasks requiring attentional behavior, which need a predefined active plan and problem‐solving skills. Errors can be associated with each type of behavior. Failures of schematic behavior are called slips or omissions and they are associated with lapses of concentration, distractions, exhaustion or burnout, or natural limitations of the human memory, for example when long lists of data fields need to be transmitted. Failures of attentional behavior are called mistakes, often occurring due to lack of experience or poor training but also arising from poor judgment, misunderstanding a situation, error when fatigue has set in, or when a process is rushed. In medicine, most errors fall in the schematic category rather than the attentional category.^20^ Checklists provide a framework to manage and reduce the risk of errors originated by slips or omissions. The aviation industry is a prime example of the successful use of checklists.^5^ The industry has learned that when pilots and air‐traffic controllers are provided with and trained in evidence‐based checklists in an environment that motivates them to follow the checklists every single time, the likelihood of errors and accidents is drastically reduced.

Checklists provide a basic memory guide and backup for those tasks that are easily forgotten and ensure that the basics are not missed (e.g., wrong patient, wrong site, missed bolus, missed electron block), allowing the team to concentrate on the more difficult and complex tasks that require more time and attention.^12^ Additionally, checklists provide a communication and workflow process that allows teams or individuals to pause, ensuring they are working together. Properly structured checklists facilitate systematic and consistent care delivery, thus reducing variability and improving performance. Checklists must have the right balance of information and structure to support clinical practice without compromising or impeding professional judgment or being overly burdensome.^21^ A risk in using checklists is the potential illusion that checklist compliance is sufficient, and everything is fine if the checklist is complete. Even the best designed and implemented checklists cannot account for all scenarios and circumstances. In medical physics, professional scrutiny while using checklists is imperative for safe use. In summary, checklists function as a supporting interface among individuals, and between individuals and their environment, helping to guide a particular workflow or procedure.^20^

## 3 CHECKLIST TEAM—QUALIFICATIONS AND RESPONSIBILITIES

Staff requirements, time allocation, and resources needed to develop and implement a checklist will scale with the scope of the checklist, as well as the size of the practice where it will be used. Development efforts can range from one individual working for a day to a large team with member representation from each clinical care group (e.g., therapist, dosimetrist, physician, nurse, physicist) working for several months.

Teamwork is an essential organizational component for a successful checklist when used in large multidisciplinary settings or where the scope of the checklist involves multiple clinical groups. As appropriate, a team approach should be used throughout all phases of development, implementation, revision, and maintenance of the checklist. Each professional group will have a varied perspective of the process and obtaining broad feedback will generate buy‐in toward future use of the checklist. There are additional incidental benefits of a multidisciplinary team approach: team members gain an improved understanding of the workflow tasks and roles as well as how work in one group impacts the others. This increased understanding may reveal opportunities for decreased duplication of efforts, increased efficiency, and improved communication. Additionally, each team member that participates during the development process acquires a sense of ownership, which will have a positive impact during implementation of the checklist into practice.

Team members who will be participating in the checklist development and implementation processes should possess the technical expertise, knowledge, and experience in the area, process, or procedure where the checklist will be used. In addition, all team members should understand the benefits of safety checklists and the goals that the checklist aims to accomplish. Members should be empowered to speak directly and honestly, thus avoiding a situation where the checklist will go unused or will hamper efficiency without improving safety. Checklists have a strong sociocultural component because they rely on individuals’ motivation, commitment, and intervention to be effective as an error prevention strategy. Therefore, an individual or group embarking on the creation of a checklist will require skills in team building and collaboration, guiding participation, conducting constructive discussions, and finding and agreeing on mutual purpose, among other management, leadership, and organizational strategies. Often, these skills are underdeveloped and are not part of any of the team members’ formal training. Some recommended literature on this topic can be found in Appendix A.

## 4 CHECKLIST GUIDELINES

### 4.1 Development and implementation processes

**FIGURE 1:**
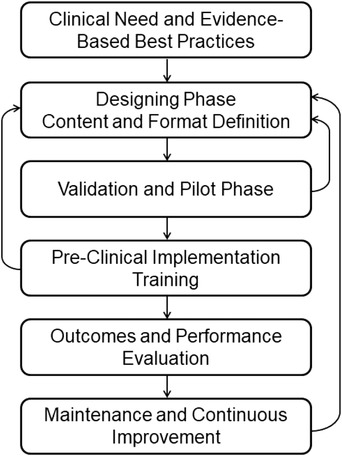
Diagram of end‐to‐end checklist development, implementation, and maintenance process

Based on current literature and best practices from aviation and medical industries^1^, ^8^, ^22^, ^23^, ^24^, ^25^ the development and implementation process can be categorized in the following steps (Figure 1).

#### 4.1.1 Clinical need and evidence‐based best practices

The first step in developing a checklist is to find specific clinical areas or processes that have the strongest evidence to improve quality and safety, and have the highest clinical impact and the lowest barriers for implementation and use.^19^ Literature review of best practices, empirical evidence, and regulatory, local and community input can help with the selection process. Examples of processes that have shown to be effective quality control checks in radiation therapy and that could benefit from checklists were presented by Ford et al.^26^ and include: physics chart review, physics weekly chart check, and therapy chart review. Additionally, high‐risk and complex procedures are examples where effective safety checklists may have high impact as an error mitigation strategy. A checklist may also be implemented as part of a corrective action in response to an incident or event.

When selecting processes or procedures that will potentially benefit from checklists, consider that an excessive use of checklists could potentially be detrimental to the practice, leading users and teams to experience “checklist fatigue”. Excessive and uncontrolled use of manual safety tools, like checklists, could make processes unnecessarily inefficient, thus decreasing the reliability of the tool and adding another layer of complexity.^22^ With this in mind, the selection process should concentrate on those tasks that are critical, often missed or overlooked, and can potentially put the patient at the highest risk for harm if not done.^27^ The checklist is a tool for task completion that does not replace professional experience and knowledge. A checklist must never be used as a strategy to resolve disciplinary issues, as a replacement for properly documented policies and procedures, or as a teaching tool by itself. However, checklists can be valuable complimentary tools to support a well‐designed educational or onboarding process.

#### 4.1.2 Designing phase—content and format definition

Poor selection or ambiguity on the checklist goal, role, or tasks will most likely lead to failure of the checklist intervention.^28^ Therefore, each checklist intervention needs to be associated with an explicit, concise, and unambiguous behavior. Methods for determining appropriate content include literature reviews, multidisciplinary focus groups, Delphi consensus,^13^ risk analysis approaches such as failure mode and effect analysis,^29^, ^30^ and causality models such as system‐theoretic process analysis.^31^ The content of the checklist should be organized so it facilitates efficient workflow. The language and sentences used for the checklist items should be simple, direct, and unambiguous, yet maintain the specialized language of the field. Checklist design should incorporate the user or team context, complementing the workflow and avoiding interference with safe and efficient care delivery. The additional time and resources needed to use and perform the checklists should be optimized and factored in the workflow. When borrowing checklists from other practices, the content and format of the checklist should not be considered absolute and will need to be evaluated and modified to fit each practice environment and workflow. Checklists should reflect up‐to‐date processes and procedures and reflect the current clinical operational context. Specific recommendations for physical checklist design are provided in Section 4.3, and example checklists are provided in Appendix C.

#### 4.1.3 Validation and pilot phase

The validation and pilot phases are essential for the success of the checklist and will help the development team detect and identify problems, risks, and issues before clinical deployment, thus avoiding complications that could lead to resistance to using the checklist.^12^, ^25^, ^32^, ^33^ This step is the first feedback loop back to the designing phase, as shown in Figure 1. In most situations, the validation of the checklist is a continuous iterative process, requiring several revisions by the development team until the checklist design is acceptable (i.e., it achieves the initial goal, and it maintains a satisfactory workflow). During the validation process, the development team works on reaching consensus on the usability, timing, potential risks, team interaction, format, and content of the checklist. After initial validation, the checklist should go through a thorough pilot testing process in a simulated clinical setup, conducted by a group representing the target individuals or team. Depending on the scale of the target group and the scope of the checklist, standard quality control methods like Plan‐Do‐Study‐Act (PDSA), as well as heuristic evaluation using interviews, focus groups, and surveys can be used during the pilot phase to collect data and improve the format and conducting method of the checklist.^23^

#### 4.1.4 Preclinical implementation training

Effective training on the use of the checklist must precede clinical deployment. Target users and teams must have a complete understanding of the purpose and methodology for using the checklist, as well as the goal of each item on the list. Simulation training using the checklist in the intended team and environment under a variety of possible scenarios should be conducted prior to clinical implementation. Consistent training should prevent misinterpretation of the items in the checklists and minimize erroneous answers or checks. During the initial time following clinical implementation, the development team should follow and monitor users and teams in clinical situations, provide guidance, and gather data to further enhance the tool. If it is discovered that the checklist contains faults or anomalies leading to common mistakes or confusion, it is important to correct the problems promptly and, if necessary, loop back to the designing stage for additional improvement of the checklist, as shown in Figure 1. The development team should seek to identify barriers to the use of the checklist.^3^, ^28^, ^34^ Section 5 identifies common barriers to checklist implementation and use, and Table B1 in Appendix B outlines strategies for overcoming common barriers.

#### 4.1.5 Outcomes and performance evaluation

Measuring performance and specific outcomes is the only way to demonstrate that the intervention—in this case, the checklist, works. It is advisable to collect baseline measurements pre‐implementation to be able to compare with post‐implementation data and evaluate and quantify the success (or failure) of the checklist. Incident reporting systems provide one method to collect this information.^17^, ^35^, ^36^ Audits of checklist compliance provide another mechanism to evaluate performance.

Ohri et al.^37^ showed that, in clinical trials, radiation therapy protocol deviations are associated with increased risk of treatment failure and overall mortality. Checklists, as an error mitigation strategy and quality assurance tool, have the potential to have an impact on clinical outcomes, but measuring this impact is very challenging and is outside of the scope of most checklist implementation processes, particularly for rare or sentinel events. Examples of achievable outcomes and end‐points that should be measured as part of a checklist implementation process include:

1. Compliance with clinical protocols, procedures, and processes.
2. Reduction of near‐misses and incidents in critical clinical processes.
3. Enhancement of communication and team dynamic.
4. Practice standardization.
5. Streamlined workflow.

Demonstrating the success of a specific checklist with concrete evidence will reinforce the utility of the tool to the group and may help motivate skeptical individuals to use the checklist.^32^

#### 4.1.6 Maintenance and continuous improvement

Checklists should evolve with practice and reflect the most current, evidence‐based data, published guidelines, end‐user feedback, and organizational changes, as well as updates on internal institutional policies, procedures, systems, machines, and instruments. As part of the practice overall quality assurance or safety program, routine reviews (e.g., annual or semi‐annual) of the practice checklists, as well as checklist performance and compliance, should be performed. Reviews should include consideration of checklist retirement if it is no longer needed to support clinical practice. Incident learning systems provide a quality control metric of the checklist performance and can flag when the tool requires further development or possibly additional training. A checklist should be considered a constantly evolving document, requiring monitoring and modifications to adapt to practice changes. Roles and responsibilities of checklist maintenance, periodic review, and continuous improvement should be clearly defined to ensure continued relevance and proper use.

### 4.2 Checklist purpose and use

How a checklist is used depends on its purpose. Some checklists guide the user through a process, preventing the omission of steps. Sometimes checklists ensure that data that will go into some process, such as a calculation, or facilitate passing information between team members, such as in a planning directive. Procedures or processes requiring multiple team members to be present at the same time (i.e., stereotactic body radiation therapy [SBRT], high dose rate [HDR] brachytherapy, stereotactic radiosurgery [SRS]), adaptive radiation therapy [ART], angiogram) might assign one person as the caller/checker of the tasks on the checklists. Upon completion of their corresponding task, the other team members will clearly state their task followed by “check” or “complete”. This approach lets the person calling the task know that the person performing the task heard the call correctly and performed the task. The most suitable method depends on the specific circumstances, the individual versus team approach, and the clinical context where the checklist will be used.

Many checklists are used to intercept possible errors—for example, evaluating a brachytherapy plan before delivery. These forms are often used by a single individual, and are most effective without the participation of the person that originally performed the task.^38^ For these forms, where appropriate and without interfering with the workflow, the person doing the check should enter the actual value from the task (such as the dose to the clinical target volume from the plan) and compare it with the corresponding limits (upper and lower limits should be included in close proximity to the relevant item on the checklist). Writing all the values helps the checker notice if the values fall outside the limits. Additionally, performance may be enhanced if the person using the checklist knows that checklist use will be audited.

The concept of redundancy is an important factor in the checklist philosophy. In any system where the human plays a central role in the outcome of a process, humans are often the weak link in the system; therefore, it is important to establish parallel redundancy to the human intervention. Based on the experience from the aviation industry, there are two types of redundancies available for the checklist use procedure. The first is between the initial configuration of a system, machine, or process and the use of the checklist as a backup only; this is called initial configuration redundancy. The second is the redundancy between team members supervising one another while conducting the checklist; this is called mutual redundancy.^24^

Checklist conducting methods can be classified into four categories:^23^

1. Static parallel or call‐do: Using this method, the checklist items are performed and completed as a series of read‐do tasks. The checklist leads the process and directs the team or individual through the process step‐by‐step. In other words, the checklist uses the “cook book” approach. This method does not use any of the redundancy strategies.
2. Static sequential with verification: This method only uses initial configuration redundancy and requires at least two individuals. One person will perform tasks from start to finish. Then, a second team member will verify each item from the checklist. This method is frequently used upon completion of a process (e.g., treatment planning) followed by the independent verification of correct completion of critical items by another team member (e.g., pretreatment plan check).
3. Static sequential with verification and confirmation: This method uses a challenge and response mechanism. During processes requiring a group approach, different members of the team perform various tasks. Upon task completion or during a reasonable procedural pause, a designated team member calls the items from the checklist and each responsible group verifies the completion and accuracy of their corresponding tasks. This method uses the combination of initial configuration and mutual redundancies as a safety barrier mechanism.
4. Dynamic: This method is suited for complex decision‐making situations, where the team is confronted with multiple options and needs to decide the optimal course of action. Emergency situations or infrequent and unpredictable critical events are suitable for the dynamic method. This method frequently uses flow charts and workflow diagrams to aid with the decision‐making process. The aviation industry uses this method for emergency and abnormal situation checklists.^25^ Arriaga et al.^39^ used this method to develop their surgical‐crisis checklists. An example of an emergency‐style checklist is shown in Appendix C, Figure C6.

A summary of the four checklists approaches, with corresponding redundancy strategies and clinical examples, can be found in Table 1.

**TABLE 1:** Checklists approaches with corresponding redundancy strategies (i.e., initial configuration redundancy or mutual redundancy)

Checklist approach	Redundancy	Example
Static parallel or call‐do	None (“cook book” approach)	Procedure to set up a water tank
Static sequential with verification	Initial configuration	Plan check process
Static sequential with verification and confirmation	Initial configuration and mutual	SBRT procedural pause
Dynamic	Initial configuration, mutual or “cook book” approach	HDR emergency procedure

*Note*: The clinical examples provide situations or processes where these approaches can be utilized.

### 4.3 Checklist design recommendations

**FIGURE C1:**
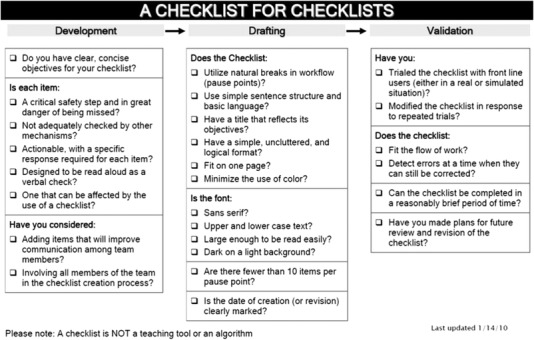
The checklist for checklists was created by Dr. Atul Gawande, the Brigham and Women's Hospital Center for Surgery and Public Health Dissemination Team, and Dan Boorman of Boeing. The goal of this checklist is to aid the checklist creation process

**FIGURE C6:**
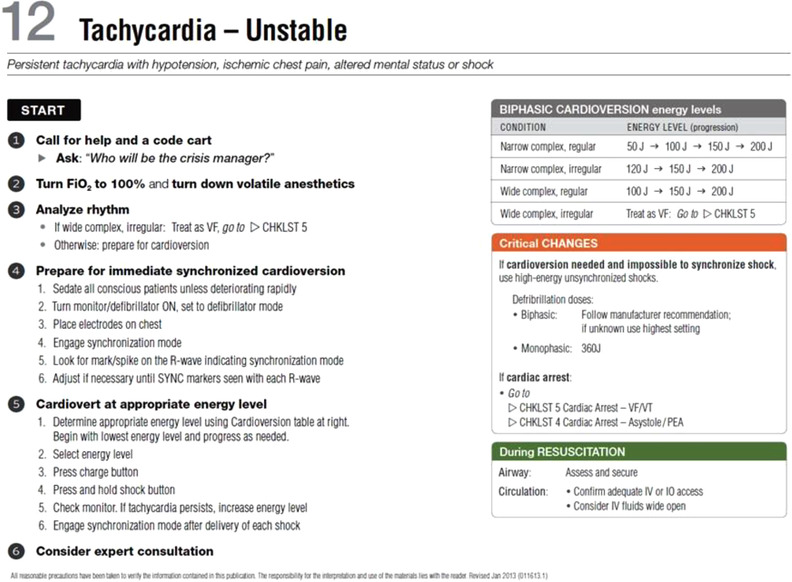
This checklist shows the differences in layout and approach when developing and using checklists for emergency or crisis situations. The actions and order of operations for the emergency procedure are on the left, and relevant data and information to facilitate rapid decision making are on the right. An example of when this format may be used in medical physics is a brachytherapy stuck source emergency. This checklist is one of the twelve checklists developed by the team at Ariadne Labs (http://www.ariadnelabs.org) with the goal of improving care during the most common operating room crises (with permission from Dr. Atul Gawande and the Safe Surgery 2015 group at Harvard School of Public Health)

The field of Human Factors Engineering uses knowledge about human characteristics, both capabilities and limitations, that are relevant during any design process and aims to optimize the interactions among people, machines, procedures, systems, and environments.^40^ There is ample evidence from both the aviation industry and the medical field showing that failing to adequately consider humans in the design and operations of systems is at best inefficient and at worst unsafe. It is important to consider applying Human Factors Engineering knowledge into the development of checklists because the checklist is a tool that relies completely on human intervention for effective performance.^41^ The following recommendations have been gathered from well‐established aviation industry guidelines^24^, ^25^ and from multiple disciplines in the medical field.^8^, ^22^, ^23^, ^41^ These recommendations can be classified into three main areas: (1) content; (2) workflow, layout, and format; and (3) physical characteristics. Additional guidance is provided in Appendix C, Figure C1. A checklist for checklists.

#### 4.3.1 Content

- A clear and unambiguous title that reflects the objective of the checklist should be defined.
- Clear guidance on the type of checklist and on what, when, and who is responsible for carrying out each of the actions and tasks in the checklist should be provided.
- Know the task and consider all task scenarios. Process mapping can facilitate understanding all the steps in the process.^29^, ^42^
- Address how the task is, or should be, performed.
- Use standard and unambiguous language and terms.
- For time‐constrained clinical situations and processes, consider the minimum number of actions that need to be included on the checklists that will provide effective and safe patient care. Iterative trial use of the checklist can help determine which actions are imperative to include while minimizing length.
- Consider the physical demands of the task and environment in which the task is being executed (e.g., subtasks to pause when hands are free, switching windows on a computer).
- Automated subtasks must be differentiated from those tasks that require attention. For an automated task, the checklist should include a check that the task is completed.
- Specific values should be recorded on the checklists if compatible with the workflow to ensure a task is not marked as ‘complete’ when the value is out of tolerance.
- The date of creation or last revision of the checklist must be clearly identified.
- All documents should identify the originator and approval route.

#### 4.3.2 Workflow, layout, and format

- Sequencing of checklist items should follow the clinical process or procedure, thus reducing the risk of users deferring checking items and potentially forgetting or missing those items and tasks.
- When compatible with the clinical process or procedure, the most critical items on the section of the checklists corresponding to that clinical process or procedure should be placed at the beginning of the section and should be completed first.
- Checklist procedures must be compatible with the operational context, restrictions, and needs of the environment where they will be used.
- Situations or processes requiring long checklists should be divided and grouped into smaller sections. Each section can be associated with systems, functions, or subprocesses. The appropriate length of checklists and subsections is highly dependent on the task and context of use; however, one should consider adding pause points or subsections at ten items or less.
- For team‐based checklists, the addition of a completion call (e.g., “checklist complete”) when the checklist is completed should be included. This step provides a cap to the checklist process and enables the team to mentally move from the checklist to other clinical operational processes and tasks.
- Natural breaks and pauses in the workflow, if such occur, should be used to perform the checklists.
- An appropriate amount of time to perform each check should be allocated as part of the clinical process or procedure. Studies show a negative relationship between the speed of performing the check and the accuracy of the check.^43^
- Standardization of the format, layout, presentation, and the checklist process should be used, especially if multiple checklists are used in a group or practice.
- Distractions and unnecessary interruptions during the performance of the checklist should be minimized.
- Fatigue (particularly mental, but also physical) should be minimized. The process should include pauses where appropriate or needed.
- The form should be quick and easy to read.
- A useful checklist must be simple but thorough.
- Use of checklists should be part of standard operating procedures of the practice.
- When compatible with the clinical process or procedure, checklist items aimed at improving the communication among team members should be included.
- Revisions to the checklist should be made as appropriate based on concerns raised by those using the checklists. For example, use of the checklist may introduce new risks. Checklists should undergo periodic review to ensure their continued applicability to the task and workflow.

#### 4.3.3 Physical characteristics

**FIGURE 2:**
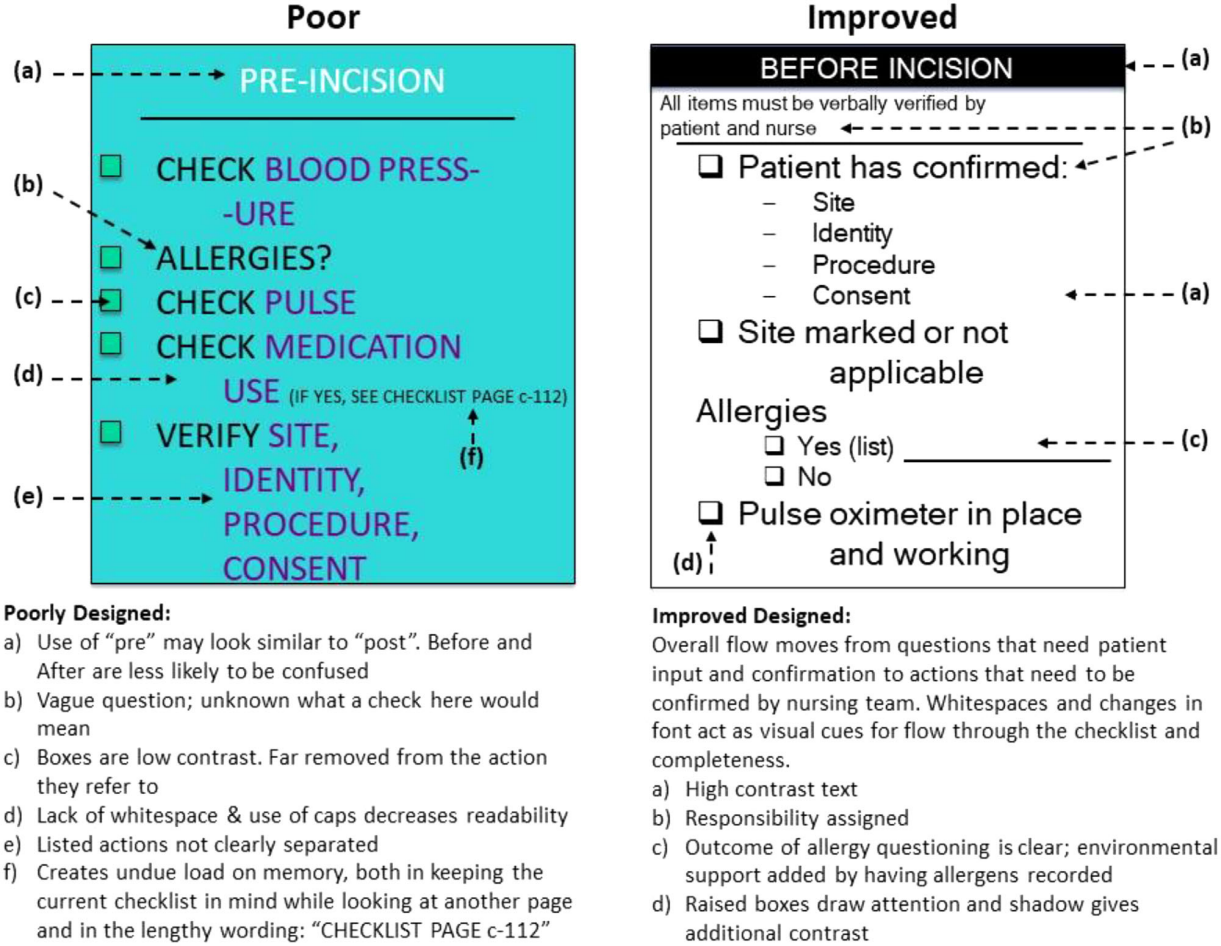
Visual comparison between a poor and an improved checklist (with permission from Dr. McLaughlin and AHRQ [Agency for Healthcare Research and Quality] Web M&M): https://psnet.ahrq.gov/perspective/what‐makes‐good‐checklist

- Font types that have clear differentiation between characters (e.g., Sans‐serif fonts, Helvetica, Gill Medium, or Arial) should be used.
- Font type should be consistent throughout the checklist.
- Lower case with upper case initial capitals should be used. Use of upper case should be limited for checklist and section headers.
- Italics for comments, notes, or supporting information are acceptable, but should be used sparingly.
- A font size that it easy to read at about arm length (60 cm) should be used (this is especially important for paper‐based checklists used under dim light conditions).
  - Font size for headings should be 14 pt (with a minimum of 12 pt).
  - Font size for normal text should be 12 pt (with a minimum of 10 pt). For cases where a checklist needs to be contained on one page, font size smaller than 12 pt may be appropriate, but must never be smaller than 10 pt.
- Black text on a white or yellow background should be used, with white text on a black background as an acceptable alternative.
- Colored text should be used with caution because of difficulties in reading colors in some lighting conditions and because of the possibility of causing confusion among colorblind individuals. Colors can be used to differentiate tasks or personnel assignments but should be used after other methods have been exhausted.
- Pastel shading can be used effectively to discriminate specific items on the checklist (e.g., cautions, consequences), but they should be used sparingly.
- The following are effective highlight methods for situations or items that require a special emphasis and differentiation, but should be used sparingly to maximize the effect:
  - Bold type.
  - Larger font size.
  - Underlining.
  - Boxing text on a white or colored background.
- Pink or red pages should not be used.
- Using some of the concepts and suggestions previously described, Figure 2 shows a visual comparison between a poor and improved checklist.^41^ Appendix C contains examples of clinical checklists use in radiation oncology, diagnostic imaging, and other areas of medicine.

#### 4.3.4 Technological considerations: Electronic and intelligent dynamic checklists

In addition to the items listed above, consideration should be given to the technical implementation of the checklist. Electronic systems have several potential advantages over paper‐based implementations including:

- Electronic interlocks such that a process or procedure cannot proceed if the checklist is not complete.
- Integration into the patient's electronic chart to facilitate communication between multidisciplinary team members.
- Formal documentation of checklist task completion.
- Ability to perform quick audits of checklist conformance.

Forward thinking about how any collected data will be used is critical, and the desire to collect data for evaluation should not supersede the length, usability, and functionality of the checklist for the intended process. Electronic signatures may enhance ownership and responsibility, potentially improving accuracy of this data. However, an electronic‐based checklist can have disadvantages when not implemented well. Electronic documents can be challenging in some electronic medical records and may tie at least one user to a computer terminal. These disadvantages are accentuated when the checklist is used in a time‐critical procedure. Electronic checklist design and implementation should therefore be approached from a sociotechnical perspective along with concepts of human‐computer interaction.^44^, ^45^ The use of simple checks, drop‐down menus, and fillable forms should follow the same design principles outlined in Section 4.3.

Intelligent dynamic checklists are a form of electronic checklists that are automatically adapted in real‐time based on pre‐programmed rules for the specific procedure or patient‐specific clinical need.^17^, ^46^, ^47^ Intelligent dynamic checklists use clinical context to maximize the relevance of the checks, can decrease the number of check items, increase checklist applicability, and reduce the absolute number of checklists in a department to minimize checklist fatigue. Intelligent dynamic checklists offer significant advantages for workflow integration; however, since many different scenarios are encoded within a single checklist, they require greater resource allocation for development and maintenance compared to static checklists. As with static checklists, it is critical that dynamic checklists are routinely updated to maintain relevance to clinical practice.

Automation can be used to facilitate and support checklists, for example out‐of‐tolerance warnings and automatically populating values to be evaluated.^48^ When automation is incorporated in the clinical workflow, it can be an effective parallel safety tool that may reduce the number of checks that must be performed.^18^, ^49^ The introduction of automation and automated checks changes the processes and associated failure modes; thus, automation may introduce new potential errors while mitigating others. When automation is introduced into a clinical process, the corresponding quality assurance and associated safety checklists should be revised, updated, and validated using formal risk analysis principles.^29^

## 5 STRATEGIES FOR SUCCESSFUL IMPLEMENTATION OF A SAFETY CHECKLIST

**FIGURE 3:**
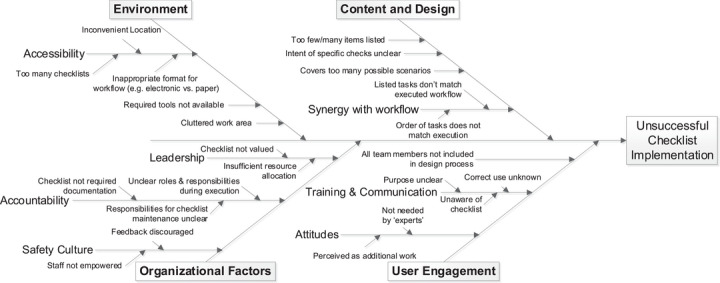
Ishikawa (cause and effect) diagram outlining common barriers to successful implementation of a checklist. Section 5 and Table B1 describe strategies to mitigate these barriers during design, training, implementation, and maintenance

Checklists can be an exceptional safety management tool, but it is critical to recognize that checklists alone cannot provide enhancements in safety and quality. Bosk et al.,^19^ in their article entitled “Reality check for Checklists”, state: “The mistake of the ‘simple checklist’ story is in the assumption that a technical solution (checklist) can solve an adaptive (sociocultural) problem.” The checklist is a supporting tool and requires convergence of many factors for effective use and ultimately for the successful completion of the associated task. Figure 3 demonstrates, using an Ishikawa (Fishbone) diagram, many of the potential barriers to successful safety checklist implementation. Prospectively addressing these barriers may ease the implementation phase and increase long term utility of the checklist tool.

### 5.1 Environment

Environmental factors that can impact checklist compliance include accessibility (location), format, number of checklists, and general work area. An inconvenient checklist location or too many checklists to choose from or to complete for a given process can deter users. Thorough consideration of the intended and actual workflow can help with identification of means for ensuring accessibility. It is important to consider checklist format (electronic vs. hard copy) with respect to accessibility and environment (e.g., lighting conditions). Clutter in the work area and unavailability of required supporting tools (e.g., pen, tablet) may also detract from effective checklist use.

### 5.2 Content and design

As detailed in Section 4, there are many considerations in the design and content of a checklist to ensure success. Importantly, the design of the checklist must not inhibit efficient completion of the task, but rather have synergy with the intended workflow by considering item order, organization, and concise language. The items on the checklist must be carefully considered so that it is not overly exhaustive but does not leave out critical or often missed components. This balance of number of items to be checked, format, and workflow can help to ensure the intention of the checklist is clear, it fits easily into the process, and its use is consistent among all team members. Thorough validation and pilot testing are critical for the development of checklists of appropriate length, content, and design for clinical use.

### 5.3 Organizational factors and safety culture

Checklists are a human‐based intervention tool, requiring a strong organizational and social infrastructure to support them, including communication, reinforcement of training, and shared knowledge. The underlying organizational component for successful implementation and effective use of safety checklists is the commitment of the department or group to establish and practice a safety culture, a safety culture is said to have four factors:^50^

- The public and private commitment of upper‐level management to safety,
- Shared attitudes towards safety and hazards,
- Flexible norms and rules to deal with hazardous situations, and
- Organizational learning.

A commonly‐cited checklist success story is the Michigan Keystone ICU (intensive care unit) program, which showed the implementation of checklists, in combination with other key elements, led to a 70% reduction of ICU‐acquired infection rates.^3^, ^51^ These key elements are:

- Summarizing, simplifying, and standardizing the process,
- Creating internal social networks with shared sense of mission and mutual reinforcement mechanisms,
- Gathering, measuring, and providing feedback on clearly defined outcomes, and
- Developing and supporting a safety culture.

Safety cultures are “characterized by communications founded on mutual trust, by shared perceptions of the importance of safety, and by confidence in the efficacy of preventive measures”.^52^ Most importantly, a safety culture is an environment where all individuals are empowered and responsible to stop a process for any safety concern without fear of consequence, ridicule, or scorn.

It is the responsibility of the practice leadership, including those with influential leadership and those with authority, to develop and maintain a safety culture, and the tools associated with that commitment. Leaders should view the development, maintenance, and use of a safety checklist as part of routine clinical duties, and they should demonstrate support through allocation of the needed time and resources. Providing a checklist to individuals and teams without building the right environment and organizational support will be a futile effort. Management and leadership support for this process is essential. Empowering staff to mutually reinforce the intended use of a safety checklist is a critical strategy for success. Team members can work together to set the normal behavior of effectively using the checklist.

Having a mechanism to hold individuals accountable for using the checklist can be helpful, especially at the initial implementation. The checklist design team, quality safety committee, or clinical leadership team should clearly identify who is expected to participate in checklist execution, who is responsible for performing the different tasks listed on the checklist, who completes the checklist, and who maintains and updates the checklist. If responsibilities are not defined, confusion and apathy can follow. If expectations, roles, and responsibilities are defined early in the implementation process, compliance issues can be corrected and coached towards less risky behavior before they solidify into habits.^53^ Celebrate early successes using the checklist, and positively recognize staff who are using the checklists successfully. These positive actions can motivate people and bring attention to the change.

### 5.4 User engagement

Just as organizational infrastructure influences the successful implementation of safety checklists, individual users need to be intrinsically motivated to use the checklist effectively. If staff do not perceive that they, the patient, or other members of the team gain benefits from the checklist, they may view the checklist as unnecessary work, or as a distraction.

User engagement barriers to checklist use include: awareness—staff may not be aware of the checklist or process; agreement—staff may not agree with items on the checklist; ambiguity—staff may not be aware of what the checklist is asking them to do; ability—staff may not have the resources, time, or skills to comply with the checklist. Strategies to mitigate common user engagement barriers are described in Appendix B, Table B1.

Experienced staff may be less keen to use a checklist than staff who are not as familiar with the procedure. Similarly, there may be skepticism about the evidence supporting the utility of checklists. It is necessary to demonstrate the need for and value of the checklist to all users as early as possible. Include experienced staff, and those who may resist the checklist, early in the checklist design process and ask them to champion the checklists in practice. During training, emphasize the intended use of the checklist (as a tool to aid staff, instead of solely documentation), and gather data about the impact of the checklist during pilot testing and routine use. Monitor the instances in which the checklist prevented tasks from being missed and communicate them to the team.

No checklist can account for all potential issues and scenarios that may arise. End users should be encouraged to continue to practice professional scrutiny and curiosity when using checklists to maximize safe and appropriate use. Checklists alone cannot provide enhancements in safety and quality, but in the appropriate organizational environment and individual user mindsets, checklists can be an exceptional safety management tool.

## 6 CONCLUSION

Effective checklists support human thinking, allow constructive team member interactions, and facilitate systematic care delivery by reducing process variability. Developing and implementing successful checklists requires a strong organizational and social infrastructure, as well as the application of well‐defined human factors engineering concepts. The guidelines presented here summarize the evidence and knowledge of the aviation industry and other medical disciplines and are aimed to guide teams and individuals in our field to develop, implement, and use checklists as a robust and effective error mitigation strategy.

## CONFLICTS OF INTEREST

The members of TG344 listed below attest that they have no potential conflicts of interest related to the subject matter or materials presented in this document. Leigh Conroy (Chair), Jacqueline T. Faught, Erika Bowers, Gillian Ecclestone, Luis E. Fong de los Santos, Annie Hsu, Jennifer Lynn Johnson, Grace Gwe‐Ya Kim, Naomi Schechter, Leah K. Schubert, and David A. Sterling. The members of the report of TG344 listed below disclose the following potential conflict(s) of interest related to subject matter or materials presented in this report.

## Figures and Tables

**FIGURE C2:**
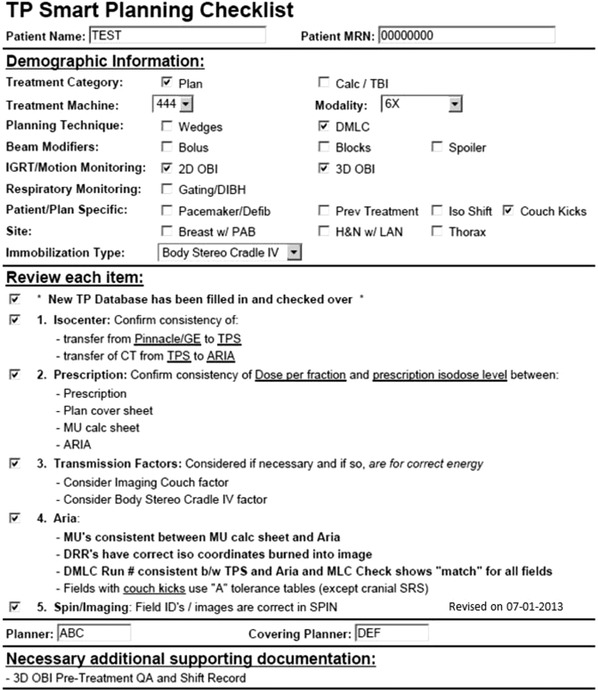
This is an example of a smart checklist tool for the treatment planning process. This tool was developed by James G. Mechalakos, PhD, Sean Berry, Ph.D, and the group at Memorial Sloan‐Kettering Cancer Center. Based on the plan demographic information and specific treatment procedure, the smart checklist tool automatically generates a custom‐built checklist. This has the benefit of avoiding items that are unrelated to the current plan, so the checklist is shorter. The tool incorporates certain forcing functions such as entering “Covering Planner” which is a required field for the checklist to be printed (courtesy of James G. Mechalakos, PhD)

**FIGURE C3:**
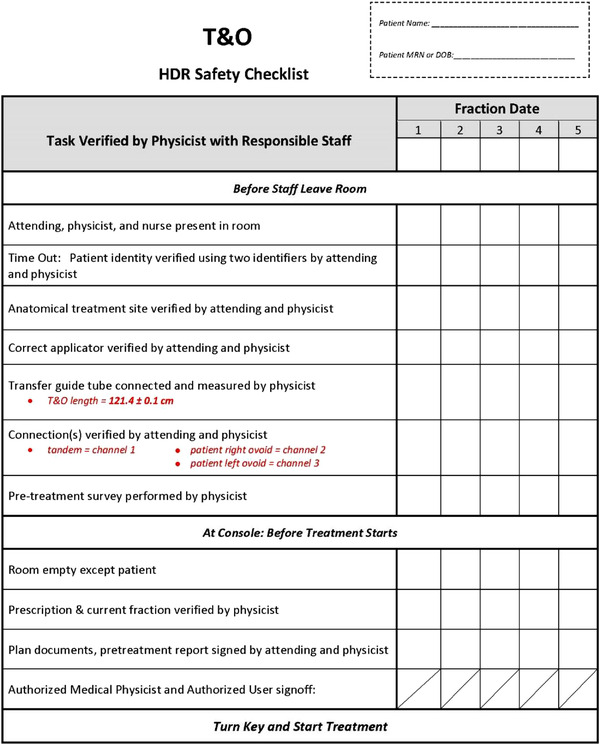
Example of a checklist used for Tandem and Ovoid (T&O) high‐dose‐rate (HDR) brachytherapy treatment from the University of Colorado (courtesy of Leah Schubert, PhD). See reference^16^ for details of checklist design and implementation

**FIGURE C4:**
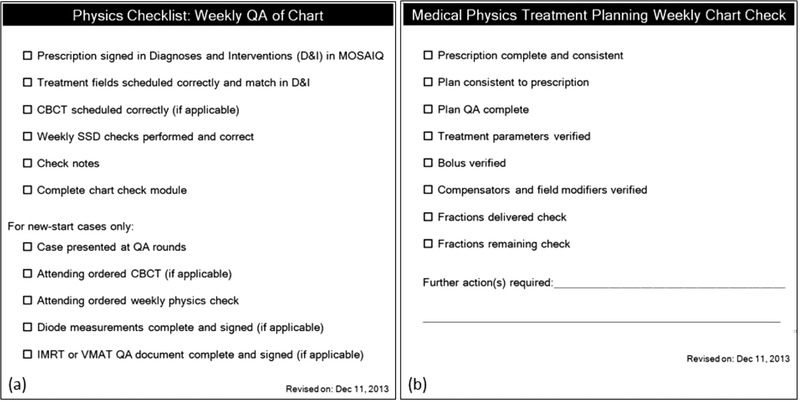
(a) Example of a checklist used as part of the physics weekly chart check process from the University of Washington (courtesy of Eric C. Ford, PhD). (b) Example of a checklist used as part of the physics weekly chart check process from Yale University Cancer Center (courtesy of Suzanne Evans, MD). Conceptually Figures 6a and 6b are achieving the same goal, nevertheless the specific elements and organization of the checklist items will depend on the clinical setting, workflow and needs of each practice. These examples emphasize the fact that each practice needs to go through their own implementation and validation process to make sure that a checklist would meet their needs

**FIGURE C5:**
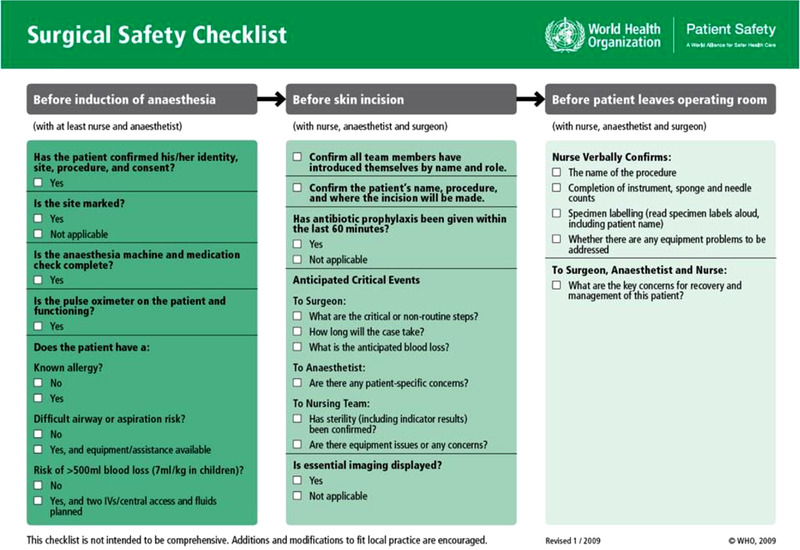
Developed by the WHO (World Health Organization) and collaborators at Ariadne Labs (http://www.ariadnelabs.org), this checklist identifies key safety steps during perioperative care that should be accomplished during every single operation no matter the setting or type of surgery. More information and details of this checklist can be found on: https://www.who.int/teams/integrated-health-services/patient-safety/research/safe-surgery. See also a ten year review of the implementation, use, and success of this checklist worldwide at: https://www.medbox.org/document/checking‐in‐on‐the‐checklist#GO (Note: this checklist example may be difficult to read for some users due to lack of contrast between the dark green background and black font in the left column. Section 4.3.3 outlines physical checklist design recommendations)

**FIGURE C7:**
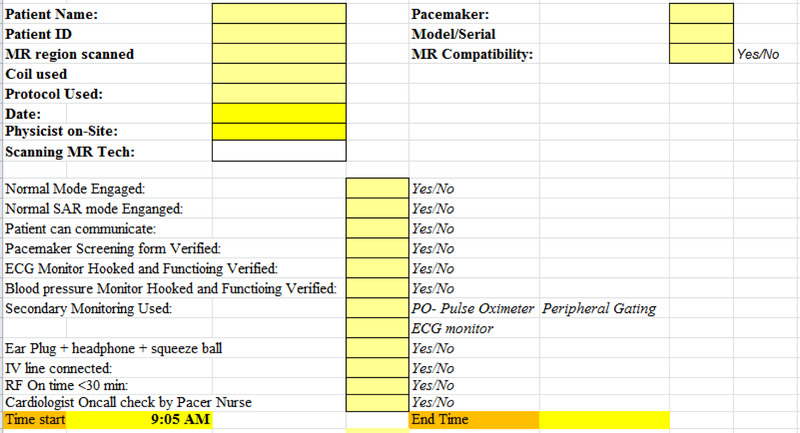
Pre‐MRI procedure medical physicist checklist. This is an example of a checklist use in diagnostic imaging. This checklist was developed by Anshuman Panda, PhD and Judy R. James, PhD from the Diagnostic Radiology group at Mayo Clinic Arizona. The goal of the checklist is to manage patients with pacemaker devices that will have an MRI procedure (courtesy of Anshuman Panda, PhD)

**FIGURE C8:**
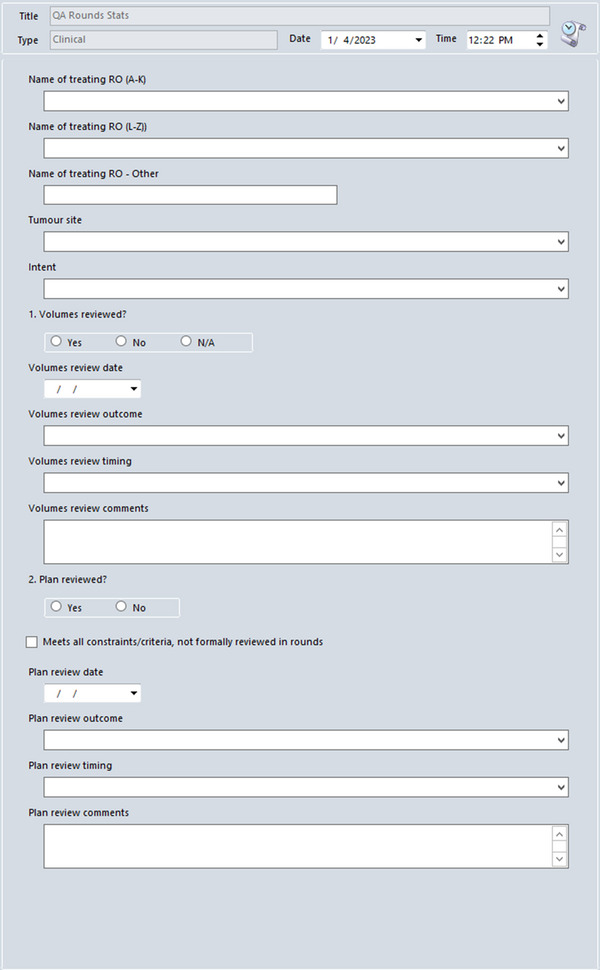
Example of an electronic checklist used to guide and collect data from multidisciplinary treatment plan peer review rounds from the Tom Baker Cancer Centre in Calgary, Alberta, Canada. (courtesy of Gillian Ecclestone, MSc)

## APPENDIX A Suggested Literature in Organization Work, Leadership, and Team Building

### A.1

1. Members of the AAPM can access leadership references and resources through the Medical Physics Leadership Academy (MPLA) at aapm.org/leadership and Quality and Safety resources at aapm.org/QualitySafety
2. Goleman D. Leadership: The power of emotional intelligence, 1st ed. More Than Sound; 2011.
3. Goleman D, Boyatzis RE, McKee A. Primal leadership: Learning to lead with emotional intelligence. Boston: Harvard Business Review Press; 2004.
4. Patterson K, Grenny J, Maxfield D, McMillan R, Switzler A. Influencer: The power to change anything, 1st ed. New York: McGraw‐Hill; 2007.
5. Patterson K, Grenny J, McMillan R, Switzler A. Crucial conversations: Tools for talking when stakes are high, 1st ed. New York: McGraw‐Hill; 2002.

## APPENDIX B Strategies to Overcome Common User Engagement Barriers to Checklist Implementation

### B.1

**TABLE B1:**

Barrier to checklist implementation	Strategies for overcoming barriers
The checklist is unclear, or gets in the way of the clinical workflow	Use a team approach when designing the checklist, and include all staff who perform the procedure. Checklist items should complement the clinical procedure and be organized for efficiency. Checklist items should focus on tasks that are critical or easily missed. Assess checklist items for brevity *and* clarity. Use simple, direct, unambiguous language yet maintain the specialized language of the field. Empower staff to speak honestly about the utility of the checklist and seek out feedback for continuous improvement. Ensure checklists and any accompanying tools (e.g., pens, tablets) are stored in easily accessible locations. Update the checklist promptly with new processes and/or changes to the clinical workflow.
No one else is using the checklist	Ensure all users receive appropriate communication, training, and opportunities for questions and feedback prior to launch. Ask leaders and experts to champion the use of the checklist. Empower staff to help each other use the checklist so that it becomes the norm. Leverage varied motivations for checklist use (e.g., evidence of improved safety, audits, improvements in team communication). Roles and responsibilities should be clearly assigned for completing the checklist items, and for maintaining the checklists.
Staff too busy with their normal work	Convince leadership to consider checklist design, use, and maintenance as part of the clinical time allocation Emphasize that the checklist is intended as a tool to aid staff and mitigate risks, as opposed to an additional hindrance Anticipate that use of checklists may increase time to complete a task and factor it into the workflow
Staff don't believe they need a checklist	Emphasize the need for and goal of the checklist. Use data whenever possible. Use a team approach when designing the checklist, and include the staff involved in the procedure, leaders, and those who might resist the checklist. Train staff on the intended use of the checklist so that staff are aware and use it correctly. Implement a reinforcement mechanism, such as audits or positive recognitions of staff using the checklist. Demonstrate early success of the checklist, such as logging and communicating times when it prevented an item from being overlooked.

## APPENDIX C Examples of Checklists

### C.1

Figures C1 to C6 are examples of checklists use in radiation oncology, diagnostic imaging, and other medical field areas, as well as a checklist for checklists created by Dr. Atul Gawande, the Brigham and Women's Hospital Center for Surgery and Public Health Dissemination Team, and Dan Boorman of Boeing. (Figures C2, C3, C4, C5, C6, C7, C8)

Additional examples can also be found at: Operating Room Crises Checklists (http://www.ariadnelabs.org/areas‐of‐work/surgery‐or‐crisischecklists/) ‐ a collection of 12 checklists to guide surgical teams during unexpected, critical events in the OR (previously known as Project Check).

Disclaimer: Checklists provided in this document must be used only as examples. We do not advise readers to apply these checklists directly to their practice, unless they perform the corresponding validation to demonstrate that they are safe and useful in their own practice.
